# Deciphering the Environmental Impacts on Rice Quality for Different Rice Cultivated Areas

**DOI:** 10.1186/s12284-018-0198-1

**Published:** 2018-01-19

**Authors:** Xiukun Li, Lian Wu, Xin Geng, Xiuhong Xia, Xuhong Wang, Zhengjin Xu, Quan Xu

**Affiliations:** Rice Research Institute of Shenyang Agricultural University, Shenyang, 110866 China

**Keywords:** Rice, Environmental factors, Quality, Dynamic deciphering

## Abstract

**Background:**

Rice (*Oryza sativa* L*.*) is cultivated in a wide range of climatic conditions, and is one of mankind’s major staple foods. The interaction of environmental factors with genotype effects major agronomic traits such as yield, quality, and resistance in rice. However, studies on the environmental factors affecting agronomic traits are often difficult to conduct because most environmental factors are dynamic and constantly changing.

**Results:**

A series of recombinant inbred lines (RILs) derived from an *indica/japonica* cross were planted into four typical rice cultivated areas arranging from latitude N22° to N42°. The environmental data from the heading to mature (45 days) stages were recorded for each RIL in the four areas. We determined that light, temperature, and humidity significantly affected the milling quality and cooking quality overall the four areas. Within each area, these environmental factors mainly affected the head rice ratio, grain length, alkali consumption, and amylose and protein content. Moreover, the effect of these environmental factors dynamically changed from heading to mature stage. Compared to light and humidity, temperature was more stable and predictable, and night temperature showed a stronger correlation efficiency to cooking quality than day temperature, and the daily temperature range had contrary effects compared to day and night temperature on grain quality.

**Conclusions:**

The present study evaluated the critical phase during the grain filling stage by calculating the dynamic changes of correlation efficiency between the quality traits and climate parameters. Our findings suggest that the sowing date could be adjusted to improve rice quality so as to adjust for environmental changes.

**Electronic supplementary material:**

The online version of this article (10.1186/s12284-018-0198-1) contains supplementary material, which is available to authorized users.

## Background

Grain yield and quality are major properties that are often investigated by agricultural scientists and breeders. Global climate change has also directly affected the environment and crop production. Several studies have shown that environmental factors had extreme effects on crop yield (Peng et al. 2004; Shi et al. 2016; Kim et al. 2016). As the living standards of society and the economy have significantly improved during the past decades, studies have focused on rice (*Oryza sativa* L.) quality traits, such as milling and cooking quality. High night temperature has been reported to decrease head rice ratio, increase chalkiness, and reduce grain width in rice (Shi et al. 2016). Rice chalkiness is a complex polygenic trait that is easily influenced by environmental conditions and certain cultural practices, particularly during the grain filling stage (Liu et al. 2010; Siebenmorgen et al. 2013). Although highly influenced by the environment, amylose content, grain length, grain width, and aspect ratio are mainly controlled by genetics (Fitzgerald et al. 2009). Poor grain quality caused by an increase in night temperature may lead to extensive reduction in economic benefits (Lyman et al. 2013).

Studies on the effects of environmental factors during the crop growth period could provide accurate information for evaluating the impact of climate on crop production. The effects of genotype, environment, and genotype × environment interaction determine the phenotypic performance and its general and specific adaptation to different environmental conditions (Balakrishnan et al. 2016). The genotype of plants can be determined through molecular and genomic approaches using DNA markers and high-throughput sequencing (Koboldt et al. 2013). However, the determination and measurement of environmental factors are relatively difficult and are largely due to two reasons. First, environmental factors are highly dynamic and constantly changing during the plant growth period, and the simple average or sum of all data cannot explain the complex effects of environmental factors. Second, environmental factors are nontrivial to be zoomed into specific individual plants so that the environmental data could be matched to the corresponding genotype. Dissecting various quantitative traits into individual Mendelian factors using molecular markers has accelerated the quantitative genetics walk out from the multiple gene investigation (Paterson et al. 1988; Lander and Botstein 1989). Dissecting complex environments into individual factors and measuring individual plants during the entire growth period may dramatically improve our understanding of the effects of environmental factors on crop production (Xu 2016). Because grain yield, quality, and stress resistance are complex traits that are affected by the environment, the selection of genotypes based on performance under a single environmental condition is inadequate for the elucidation of the effect of an environmental factor on crop production. An investigation based on a multi-environment may provide more information of the effects of environmental factors on crop production.

The main objective of the present study was to dynamically and constantly decipher the effects of environmental factors on quality traits of rice under different environmental conditions.

## Results

### Assessment of Environmental Dactors in Four Areas

**Fig. 1:**
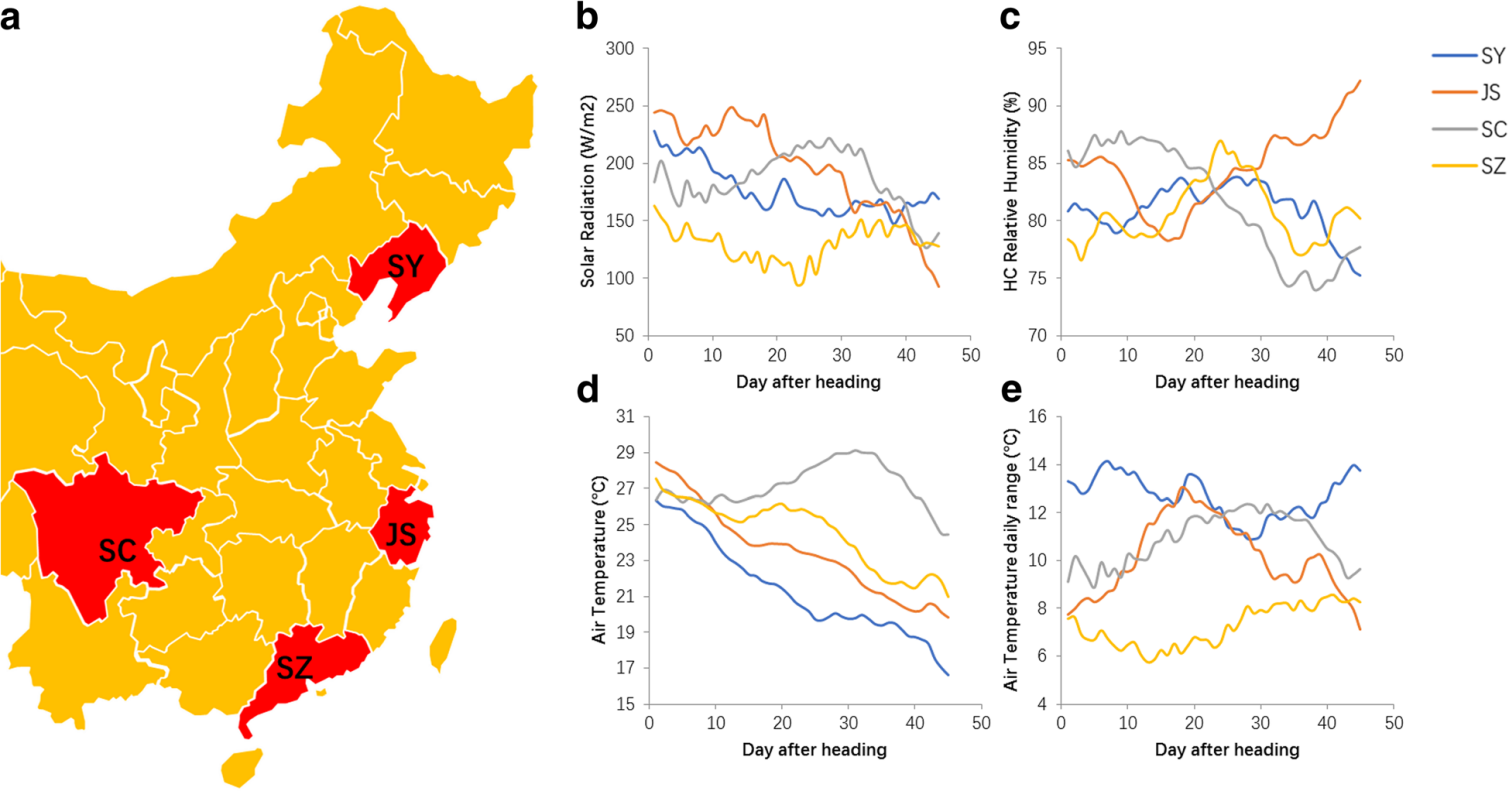
The main environmental factors of the four cultivated areas. **a** The position of four typical rice cultivated areas, **b**-**e** The data from 45 days of monitoring various environmental factors in four areas

To determine the effect of various environmental factors on rice quality, we planted a series of 155 recombinant inbred lines (RILs), derived from a cross between *indica* variety “R99” and *japonica* variety “SN265” into four typical rice cultivate areas, SY (N42°), JS (N32°), SC (N31°), and SZ (N22°). The location of the cultivated area is shown in Fig. 1a. The survey of environmental data included light, temperature, and humidity was conducted for nearly 3 months at all four areas. Because the 155 lines have different heading dates, the environmental conditions of 45 days after heading were very different for each line (Additional file 1: Table S1). Thus, the plants underwent diverse environmental conditions from the heading stage to the mature stage even if they were cultivated in same area. In the present study, we selected the environmental data of day 1 after heading for 155 lines in SY, then used the average data to represent the environmental condition for day 1 after heading in SY. Then, we collected data from day 1 to day 45 after heading using this method. These data represented the environmental conditions which plants really experience from the heading stage to the mature stage in SY. The environmental data of the 45-day from heading of each line in four areas were collected to conduct the following analysis. The results showed that the environmental conditions that RILs experienced during those 45 days were highly diverse (Fig. 1). The air temperature was more stable and predictable than were light and humidity, and the air temperature was ranked as SC > SZ > JS > SZ (Fig. 1d). SY had a high daily temperature range during the 45 days of monitoring, SC and JS showed a similar daily temperature range as that of SY for 20–30 days, and SZ had a low daily temperature range (Fig. 1e). In terms of solar radiation, SY and JS experienced more radiation than did SC and SZ during the earlier stage; for SC, it increased at the later stage, and SZ received the least solar radiation during the entire study (Fig. 1b). Humidity showed the opposite patterns as those observed for solar radiation (Fig. 1c).

### Quality Measurement of RILs in the Four Areas

**Fig. 2:**
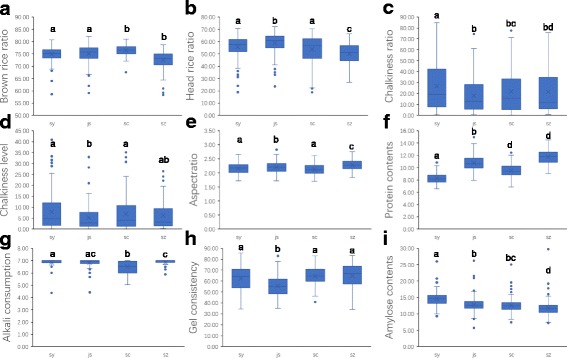
The box-plot graph of the rice quality traits of RILs in four cultivated areas. **a** brown rice ratio, **b** head rice ratio, **c** chalkiness rice ratio, **d** chalkiness level, **e** grain length/grain width, **f** protein content, **g** alkali consumption, **h** gel consistency, and **i** amylose content. Letter difference means significant at 5% probability levels by Duncan’s new multiple range method

We conducted a quality measurement, including brown rice ratio, milled rice ratio, head rice ratio, chalkiness rice ratio, chalkiness level, grain length, grain width, alkali consumption, gel consistency, amylose content, and protein content of the RILs in the four areas upon reaching maturity. Our results showed extensive variations in quality traits among the four areas. All traits exhibited significant differences among the four areas except for brown rice ratio, milled rice ratio, and alkali consumption between SY and JS, and the chalkiness rice ratio, chalkiness level, and gel consistency between SC and SZ (Fig. 2 and Table 1). We subsequently compared variations that may have been caused by differences in environmental conditions and genetic diversity. The maximum data minus the minimum data of the 155 lines in one area represent the variation caused by genotype. The maximum data minus the minimum data among the four areas for an individual line represent the variation caused by environmental conditions. The results showed that the environmental conditions have a stronger effect on protein content and alkali consumption than genotype, and genotype has stronger impact on amylose content and grain width than environmental factors (Table 1).

**Table 1:** The Quality Traits of RILs in Four Areas

Area		Brown rice (%)	Milled rice (%)	Head rice (%)	Chalkiness ratio (%)	Chalkiness level	Grain length (mm)	Grain width (mm)	Aspect ratio (Grain length/width)	Alkali consumption	Gel consistency	Amylose content	Protein content
SY	Average	74.944 ± 2.999^a^	67.443 ± 2.707^a^	55.748 ± 9.095^a^	26.551 ± 23.168^a^	7.875 ± 8.283^a^	5.436 ± 0.255^a^	2.536 ± 0.183^a^	2.156 ± 0.199^a^	6.869 ± 0.295^a^	62.421 ± 12.849^a^	14.517 ± 2.035^a^	8.263 ± 0.817^a^
	Max-Min	22.150	19.930	51.625	84.350	40.850	1.425	0.900	0.941	2.625	59.391	16.710	4.585
JS	Average	75.043 ± 3.729^a^	67.559 ± 3.383^a^	58.806 ± 8.367^b^	18.262 ± 15.993^b^	5.197 ± 5.507^b^	5.386 ± 0.266^b^	2.458 ± 0.191^b^	2.203 ± 0.197^b^	6.826 ± 0.303^b^	55.407 ± 9.634^b^	12.851 ± 2.523^b^	10.798 ± 1.26^b^
	Max-Min	23.073	20.770	48.525	73.925	32.900	1.575	0.950	1.108	2.583	47.680	20.509	7.255
SC	Average	76.513 ± 2.015^b^	68.861 ± 1.817^b^	53.623 ± 11.742^c^	21.7 ± 19.238^c^	6.848 ± 7.211^c^	5.437 ± 0.226^ac^	2.573 ± 0.198^c^	2.121 ± 0.191^c^	6.505 ± 0.424^ac^	64.532 ± 8.497^c^	12.548 ± 2.214^c^	9.51 ± 1.017^c^
	Max-Min	8.870	7.982	51.775	77.425	35.100	1.250	0.975	0.913	1.975	41.649	17.669	5.610
SZ	Average	72.413 ± 3.753^c^	65.167 ± 3.378^c^	50.064 ± 8.772^d^	21.572 ± 20.382^cd^	6.241 ± 6.544^cd^	5.514 ± 0.237^d^	2.434 ± 0.168^d^	2.275 ± 0.182^d^	6.874 ± 0.223^d^	64.964 ± 11.488^cd^	11.73 ± 2.333^d^	11.748 ± 1.251^d^
	Max-Min	20.343	18.305	39.325	74.825	26.950	1.225	0.825	1.046	1.125	50.853	22.530	5.500
Individual	Max-Min	15.612 (RIL65)	14.0475 (RIL65)	33.200 (RIL141)	58.650 (RIL54)	33.800 (RIL119)	1.300 (RIL144)	0.550 (RIL150)	0.463 (RIL126)	2.630 (RIL112)	37.504 (RIL133)	8.220 (RIL68)	7.935 (RIL98)

Letter difference means significant at 5% probability levels by Duncan’s new multiple range method

### Relationship Between Environmental Factors and Rice Quality

We subsequently divided light factor into solar radiation, lux meter, and light hour, divided air temperature into whole day average air temperature, day temperature, night temperature, and daily temperature range, and divided relative humidity into whole day average humidity, day humidity, and night humidity, generating a total of 10 parameters. Because each line had specific 45 days of environmental data, and each line also had a value of quality traits, the correlation analysis could be conducted between environmental data and quality traits. The present analysis was based on a one-on-one relationship between a specific environmental factor and particular individual line. Then, we conducted a correlation analysis between quality traits and the 10 parameters. In its entirety, the 10 parameters were significantly correlated with almost all quality traits except for chalkiness traits. Temperature showed a weaker correlation with grain shape traits than did light and humidity. Within each area, these 10 parameters exhibited a weaker correlation with brown rice ratio, milled rice ratio, and gel consistency than over all four areas together. The 10 parameters showed weaker correlation with quality traits in SZ than that in the other three areas (Table 2).

**Table 2:** The Correlation Efficiency Between Quality Traits and Environmental factors

Area	Environmental factors	Brown rice	Milled rice	Head rice	Chalkiness grain	Chalkiness level	Grain length	Grain width	Aspect ratio	Alkali consumption	Gel consistency	Amylose contents	Protein contents
Entirety	Solar radiation	0.286**	0.285**	0.166*	0.056	0.072	−0.254**	0.109*	−0.221**	−0.188**	−0.158**	0.063	−0.294**
	Lux meter	0.246**	0.243**	0.017	0.085	0.108*	−0.201**	0.127*	−0.208**	−0.293**	0.015	−0.066	−0.196**
	Light hour	0.359**	0.357**	0.112*	0.077	0.097	−0.196**	0.223**	−0.289**	−0.257**	−0.035	0.142**	−0.518**
	Air temperature	0.066	0.065	−0.194*	0.003	0.033	−0.057	0.035	−0.063	−0.363**	0.154**	−0.386**	0.320**
	Day temperature	0.130**	0.128**	−0.170*	0.017	0.049	−0.074	0.083	−0.112*	−0.401**	0.153**	−0.335**	0.196**
	Night temperature	−0.01	−0.012	−0.221**	−0.015	0.01	−0.015	−0.012	−0.002	−0.306**	0.157**	−0.420**	0.438**
	Daily range	0.297**	0.295**	0.181**	0.088	0.089	−0.109*	0.219**	−0.238**	−0.078	−0.055	0.364**	−0.733**
	Relative humidity	0.107*	0.112*	0.144**	−0.095	−0.079	−0.129*	−0.064	−0.013	0.019	−0.271**	−0.017	0.160**
	Day humidity	−0.189**	−0.184**	0.009	−0.118*	−0.119*	−0.015	−0.238**	0.192**	0.195**	−0.191**	−0.187**	0.584**
	Night humidity	0.282**	0.283**	0.246**	0.021	0.028	−0.171**	0.122*	−0.189**	−0.029	−0.217**	0.286**	−0.486**
SY	Solar radiation	−0.055	−0.054	−0.258**	0.149	0.160*	−0.246**	−0.058	−0.08	−0.315**	0.071	−0.334**	0.163*
	Lux meter	0.029	0.029	−0.213**	0.128	0.137	−0.209**	−0.021	−0.096	−0.273**	0.063	−0.285**	0.161*
	Light hour	−0.133	−0.132	−0.279**	0.139	0.156	−0.218**	−0.032	−0.079	−0.221**	0.051	−0.325**	0.174*
	Air temperature	−0.066	−0.066	−0.280**	0.152	0.156	−0.234**	−0.055	−0.077	−0.319**	0.076	−0.346**	0.156
	Day temperature	−0.064	−0.064	−0.276**	0.15	0.15	−0.235**	−0.055	−0.077	−0.317**	0.075	−0.346**	0.159*
	Night temperature	−0.07	−0.069	−0.286**	0.154	0.154	−0.233**	−0.055	−0.076	−0.321**	0.077	−0.346**	0.152
	Daily range	0.124	0.124	0.192*	−0.137	−0.135	0.131	−0.005	0.071	0.254**	−0.025	0.188*	−0.065
	Relative humidity	−0.068	−0.068	−0.303**	0.147	0.158	−0.212**	−0.058	−0.064	−0.304**	0.093	−0.346**	0.102
	Day humidity	−0.074	−0.074	−0.309**	0.148	0.157	−0.202*	−0.059	−0.058	−0.301**	0.094	−0.333**	0.089
	Night humidity	−0.051	−0.051	−0.277	0.137	0.152	−0.225**	−0.052	−0.076	−0.300**	0.088	−0.362**	0.128
JS	Solar radiation	−0.1	−0.11	−0.181*	0.101	0.396**	−0.343**	−0.119	−0.091	−0.176*	0.161*	−0.470**	0.434**
	Lux meter	−0.103	−0.123	−0.161*	0.1	0.149	−0.350**	−0.113	−0.097	−0.184*	0.165*	−0.464**	0.426**
	Light hour	−0.086	−0.128	−0.147	0.095	0.188*	−0.375**	−0.118	−0.108	−0.151	0.156	−0.452**	0.420**
	Air temperature	−0.1	−0.111	−0.180*	0.097	0.128	−0.353**	−0.118	−0.097	−0.134	0.157	−0.460**	0.423**
	Day temperature	−0.099	−0.11	−0.178*	0.098	0.11	−0.353**	−0.117	−0.098	−0.14	0.159*	−0.462**	0.427**
	Night temperature	−0.102	−0.113	−0.183*	0.095	0.144	−0.353**	−0.119	−0.096	−0.123	0.153	−0.454**	0.415**
	Daily range	−0.114	−0.124	−0.053	0.154	0.103	−0.241**	−0.058	−0.086	−0.131	0.131	−0.357**	0.369**
	Relative humidity	0.08	0.091	0.168*	−0.158	−0.152	0.312**	0.1	0.09	0.165*	−0.161*	0.437**	−0.420**
	Day humidity	0.078	0.118	0.163*	−0.115	−0.270**	0.304**	0.096	0.09	0.171*	−0.162*	0.441**	−0.426**
	Night humidity	0.102	0.093	0.182*	−0.142	−0.361**	0.334**	0.116	0.086	0.126	−0.149	0.384**	−0.365**
SC	Solar radiation	−0.048	−0.047	−0.399**	0.117	0.212**	−0.135	0.074	−0.159	0.224**	0.138	−0.199*	0.353**
	Lux meter	0.104	0.105	−0.088	0.063	0.061	−0.142	−0.033	−0.027	0.055	0.039	−0.027	0.062
	Light hour	−0.109	−0.109	−0.386**	0.170*	0.171*	−0.185*	0.057	−0.174*	0.221**	0.176*	−0.219**	0.419**
	Air temperature	−0.09	−0.091	−0.390**	0.158	0.144	−0.206*	0.042	−0.157	0.215**	0.132	−0.169*	0.454**
	Day temperature	−0.072	−0.073	−0.393**	0.168*	0.151	−0.187*	0.052	−0.156	0.219**	0.121	−0.156	0.422**
	Night temperature	−0.109	−0.11	−0.376**	0.141	0.131	−0.225**	0.029	−0.153	0.203*	0.142	−0.180*	0.479**
	Daily range	0.096	0.098	−0.119	0.079	0.052	0.05	0.073	−0.044	0.107	−0.047	0.059	−0.028
	Relative humidity	−0.14	−0.14	−0.319**	0.186*	0.206*	−0.190*	0.036	−0.15	0.161*	0.211**	−0.275**	0.421**
	Day humidity	−0.147	−0.149	−0.280**	0.153	0.176*	−0.193*	0.019	−0.133	0.137	0.203*	−0.269**	0.418**
	Night humidity	−0.122	−0.122	−0.348**	0.216**	0.231**	−0.185*	0.046	−0.159*	0.183*	0.211**	−0.269**	0.404**
SZ	Solar radiation	−0.043	−0.153	0.095	0.084	0.014	0.102	0.019	0.019	0.024	0.113	−0.153	0.058
	Lux meter	−0.162*	−0.162*	0.082	0.053	0.081	−0.043	−0.045	0.004	−0.042	−0.147	0.104	−0.228
	Light hour	0.142	0.142	0.121	−0.062	−0.077	−0.164*	−0.114	0.025	0.071	0.105	−0.152	0.240**
	Air temperature	0.103	0.103	0.162*	−0.062	−0.074	−0.193*	−0.131	0.028	0.055	0.043	−0.160*	0.207*
	Day temperature	0.094	0.094	0.169*	−0.059	−0.067	−0.194*	−0.132	0.028	0.058	0.035	−0.149	0.198*
	Night temperature	0.108	0.109	0.153	−0.063	−0.079	−0.187*	−0.129	0.03	0.053	0.049	−0.169*	0.215**
	Daily range	−0.163*	−0.163*	−0.021	0.046	0.08	0.034	0.049	−0.039	−0.054	−0.134	0.14	−0.228**
	Relative humidity	−0.052	−0.054	−0.056	0.051	0.085	0.208**	0.113	0.003	0.062	−0.032	0.193*	−0.132
	Day humidity	0.027	0.025	−0.077	0.028	0.042	0.212**	0.119	0.007	0.081	0.038	0.128	−0.021
	Night humidity	−0.098	−0.099	−0.035	0.061	0.103	0.174*	0.096	−0.004	0.037	−0.075	0.210**	−0.190*

*P < 0.05, **P < 0.01

Protein content showed a significant positive correlation with whole day average temperature, and the protein content of plants grown in SY, JS and SZ could be ranked as SY < JS < SZ. However, SC, which had the highest temperature, had protein content that were only higher than that of SY. This may be explained by changes in solar radiation and humidity in SC. Solar radiation increased from 10 days after heading and was highest at 20–30 days, whereas a negative correlation between solar radiation and protein content was observed in this stage. Humidity significantly decreased after 20 days and was significantly correlated with protein content during this stage. Consequently, changes in solar radiation and humidity in SC may have impaired the increase of protein content that was caused by high temperature.

In order to elucidate the interaction between each environmental factor to rice quality, we conducted a path analysis as shown in Additional file 1: Table S2. For milling quality, the indirect path coefficients from solar radiation through daily temperature range to brown rice ratio and milled rice ratio were 0.423 and 0.399, respectively. The indirect path coefficients from solar through daily temperature range were 0.423 and 0.399, respectively. For appearance quality, the indirect path coefficient from solar through daily temperature range was −0.245. For cooking quality, the indirect path coefficients from solar through daily temperature range to alkali consumption and amylose content were −0.236 and 0.422, respectively. For nutritional quality, the indirect path coefficients from solar through daily temperature range to protein content was −0.843. For amylose content and protein content, the indirect path coefficient from relative humidity through solar radiation also reached −0.236 and 0.335, respectively. The indirect path coefficients among environmental factors were at a low level in head rice ratio and gel consistency.

### Dynamic analysis of Environmental Factors to Rice Quality

**Fig. 3:**
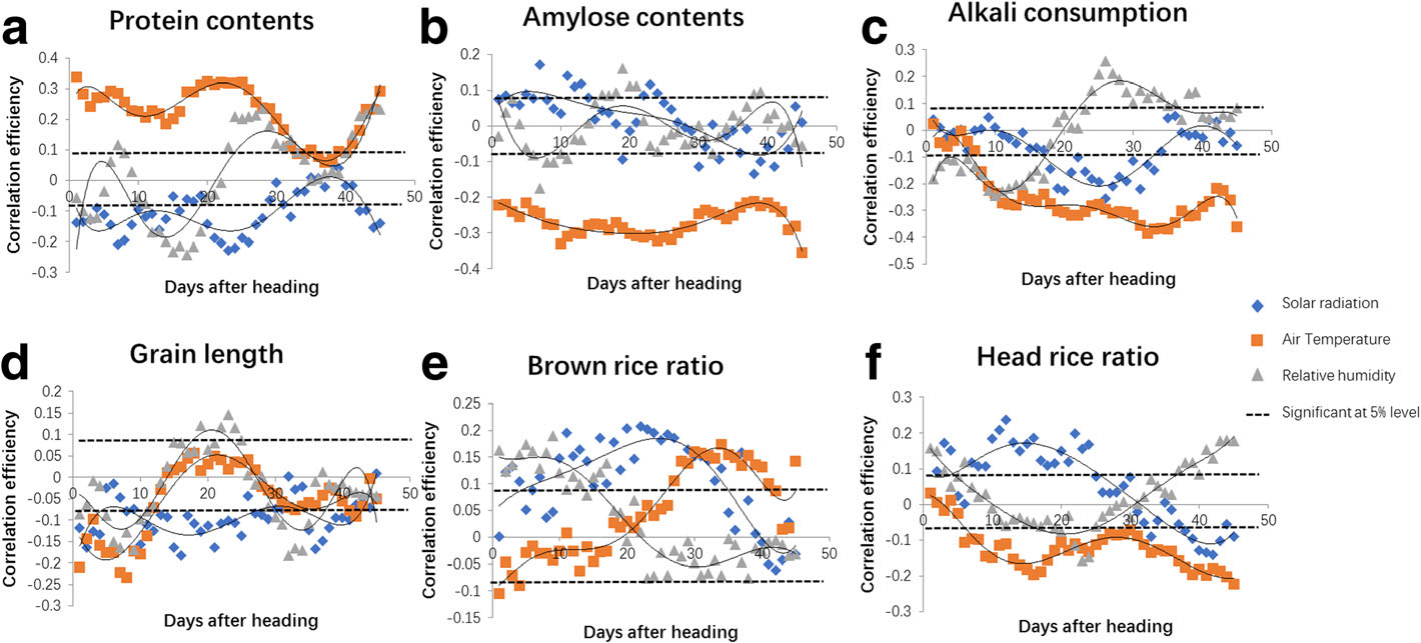
The dynamic analysis of the correlation of environmental traits to quality traits. The correlation efficiency of environmental factors (solar radiation, whole day average temperature, and relative humidity) to **a** protein content, **b** amylose content, **c** alkali consumption, **d** grain length, **e** brown rice ratio, and **f** head rice ratio

**Fig. 4:**
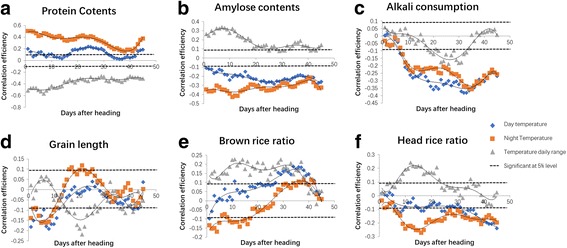
The dynamic analysis of the correlation of temperature factors to quality traits. The correlation efficiency of temperature factors (day average temperature, night average temperature, and daily temperature range) to **a** protein content, **b** amylose content, **c** alkali consumption, **d** grain length, **e** brown rice ratio, and **f** head rice ratio

As environmental factors are dynamic and constantly change, we conducted a dynamic analysis of the environmental factors on quality to elucidate the effects of changes in environmental factors from the heading stage to the mature stage. The results showed that the effect of environmental factors became stronger or weaker over time (Fig. 3). Compared to light and humidity, the effect of temperature on rice quality traits was more stable and predictable. The whole day average temperature showed a significant positive correlation with protein content and a significant negative correlation with amylose content, alkali consumption, and head rice ratio during the entire 45-day survey; it had a negative correlation with brown rice ratio in the first 20 days, but subsequently exhibited a positive correlation (Fig. 3). Interestingly, the daily temperature range exhibited the opposite effects compared to the whole day average temperature, particularly in terms of protein content, amylose content, grain length, and head rice ratio (Fig. 4). Day temperature and night temperature showed similar effects as that of whole day average temperature, whereas the night temperature exhibited a stronger correlation with protein content, amylose content, and head rice ratio. The effects of light and humidity on quality traits showed weaker regularity compared to temperature. Solar radiation exhibited a negative correlation with protein content, alkali consumption, and grain length during the entire 45-day survey. Solar radiation was found to have a positive correlation with brown rice ratio and head rice ratio during the early and middle stages, yet a negative correlation was observed at the later stage. Lux meter and light hour showed similar effects on quality traits as that of solar radiation (Additional file 1: Figure S1). Day and night humidity also exhibited similar correlations with rice quality traits as that of whole day average humidity (Additional file 1: Figure S2).

## Discussion

Agronomic traits such as yield, quality and stress resistance are mainly determined by the interaction between genotype and environment. Genotypes are generally assessed using DNA markers and high-throughput sequencing (Koboldt et al. 2013). Measuring the impact of the environment has largely been considered as a single factor that interacts with the particular genotype of a specific plant. As plants grow in a constantly changing environment, plants with different growth periods are subjected to variations in environmental conditions even when they are cultivated in the same area. Thus, investigations on the impact of environmental factors on crop production of specific plants are warranted.

The previous studies showed that environmental factors strongly affect rice quality. In the filling stage, the head rice ratio decreased 24%–35% when the temperature increased from 20 to 30 °C (Ha et al. 1994). The air temperature has been reported to have a negative correlation with amylose content (Asaoka et al. 1985), and was the major factor effecting protein content in rice during the period of 10–20 days after heading (Nakamura et al. 1989). However, few studies simultaneously investigated the effects of solar radiation, temperature and humidity on rice milling quality, appearance quality, nutritional quality and cooking quality. The present study integrated the data of solar radiation, air temperature, daily temperature range and relative humidity, with the goal of elucidating the interaction between each environmental factor and RIL lines on rice quality. The path analysis results showed that solar radiation and daily temperature range have a significant correlation with almost all quality traits. Also, the indirect path coefficient from solar radiation through daily temperature range to quality traits were at a high level. For amylose content and protein content, the indirect path coefficient from solar radiation through relative humidity also reached a high level. These results indicated that solar radiation and daily temperature range may have an interaction, and play a major role in effecting quality traits. The relative humidity may participate in this interaction to affect amylose content and protein content. Moreover, the present study determined that the effects of environmental factors on quality traits dramatically change from the heading to the mature stage. For example, the air temperature was negatively correlated with the brown rice ratio before 20 days, but was significantly positively correlated after 20 days. Humidity showed a significant negative correlation with head rice ratio in the mid stage, whereas a significant positive correlation with head rice ratio was observed at the earlier and later stages. In summary, environmental factors are dynamic and constantly changing; therefore, simply calculating the average data of environmental factors may not precisely explain their effects on quality traits. The results of our study suggest that the appropriate adjustments to the sowing date should be made to cater to or avoid to the corresponding advantages or disadvantages of specific environmental conditions.

An investigation of the years from 1951 to 2010 has observed an asymmetric warming, with greater increase in night than in day temperature (Donat and Alexander 2012). The annual mean day temperature and night temperature increased by 0.35 °C and 1.13 °C, respectively (Peng et al. 2004). Such an increase in air temperature will profoundly affect crop production, and many studies show a significant influence of elevated temperature on crop yield. Grain yield declined by 10% for each 1 °C increase (Peng et al. 2004). Grain quality in most hybrids was also strongly affected by high night temperature, decreased head rice ratio, increased chalkiness, and reduced grain width (Shi et al. 2016). A meta-analysis demonstrated that high temperature affected grain quality by decreasing the head rice percentage and increasing the chalkiness rice ratio and chalkiness level (Xiong et al. 2017). In our study, we also found that night temperature more severely affected quality traits than day temperature. Moreover, we found that the daily temperature range is an important parameter that affects quality as it has the opposite effect on quality traits compared to day and night temperature. The whole day average temperature has a significant negative correlation with amylose content, but the daily temperature range showed a positive correlation with amylose content. The lowest whole day average temperature and the largest daily temperature range in SY resulted in the highest amylose content. The smallest temperature range in SZ resulted in the lowest amylose content despite the lower whole day average temperature relative to that of SC.

The environmental factors strongly affected the yield components. However, we only conducted a 45-day survey of the environmental factors, and thus, we were unable to evaluate the effect of environmental factors on heading date, plant height, and various yield components. The effect of various environmental factors on the entire growth period should be investigated in a future study. We found that some RILs showed extremely large variations among four areas, whereas only a few RILs exhibited a stable phenotype in all four areas. The molecular mechanism underlying the stability of a phenotype among different areas requires further investigation. The stable lines may also serve as a rare germplasm for breeding highly adaptable varieties that exhibit elite phenotypic features in different areas.

## Conclusions

The precise dissection of complex environmental factors for specific individual plants provides us with a novel strategy for the optimization of environmental factors for crop quality improvement. In the present study, we monitored various environmental factors surrounding 155 RILs that were planted in four areas for 45 days to decipher their impact on rice grain quality. The results showed that environmental factors had variable effects on rice grain quality, ranging from the heading stage to the mature stage. Based on our findings, we suggest that field management adjust the sowing date to cater to the current environmental conditions in order to improve rice quality.

## Method

### Plant Materials

A total of 155 RILs derived from a cross between ‘Shennong265’ (*Oryza. sativa L. ssp. japonica*) and ‘R99’ (*Oryza. sativa L. ssp. indica*) was used in this study. This RIL population was developed from a single-seed descendant that had been inbred for over 10 generations. Field experiments were conducted in four typical rice cultivated areas: the Rice Research Institute of Shenyang Agricultural University (SY)(N41°, E123°), the sub base of China National Hybrid Rice R&D Center in Jiangsu Province (JS N32°, E120°), the Academy of Agricultural Sciences of Sichuan Province (SCN32°, E104°), and the Agricultural Genomics Institute at Shenzhen (SZN22°, E114°) for two growing seasons during 2015–2016. At SY, the seeds were sown on April 15 and transplanted on May 21. At JS, they were sown on May 8 and transplanted on June 8. At SC, they were sown on April 12 and transplanted on May 9. At SZ, they were sown on July 20 and transplanted on August 12. Seeds were sown in both 2015 and 2016. Three rows were planted for each line, with 10 plants per row, and a plant spacing of 30 cm × 13.3 cm. The RILs were arranged in a randomized block design with two replicates. Cultivation methods and field management varied according to regional cultivation practices. The paddies were tested before sowing, and the fertility of soil in the four areas is shown in Additional file 1: Table S3. Fertilizers were applied with a basal dressing amount of 150 kg N per hectare, 150 kg P per hectare and 150 kg K per hectare. We added 75 kg N per hectare 7 days after transplanting as a top application. The paddies were harvested at 45 days after heading for each line in each of the four areas.

#### Quality Measurements

Mature rice grains were milled after harvest, air dried, and stored at room temperature for 3 months. A total of 20 plants from the middle rows were harvested for each line. The brown rice ratio, milled rice ratio, and head rice ratio were calculated after harvest. The grains were dehulled to produce brown rice using Rubber Roll Sheller (THU testing hunsker, Satake, Hiroshima, Japan), and brown rice ratio was determined. Thereafter, the brown rice was milled with rice-polishing machine (TM05 test mill, Satake). After milling, head rice and broken rice were separated and finally, the milled rice ratio and head rice ratio were expressed as percentage of total weight of rough rice. Amylose content and gel consistency were assessed according to The National Standard of the People’s Republic of China (GB/T17891–1999). Extraction and measurement of rice protein compositions was performed as described by Li et al. (2000) and Tan et al. (1999). All samples were analyzed twice. As the 2 years of data are highly correlated and showed similar trends the 2016 data was used in the subsequent analysis (Additional file 1: Table S4).

### Assessment of Environmental Factors and Data Analysis

The environmental factors were measured using iMETOS (Pessl Instruments GmbH, Weiz, Austria) at four areas. We surveyed three main environmental factors: light, which was subsequently divided into solar radiation, lux meter, and light hours; temperature, which includes whole day average air temperature, day air temperature, night air temperature, daily and temperature range; and humidity, which includes whole day average relative humidity, day relative humidity, and night humidity. The survey was conducted for nearly 3 months at all four areas. The environmental data of the 45-day from heading for each line in four areas were collected to conduct the analysis. The data were analyzed using SPSS 17.0.

## Additional File

Additional file 1: Figure S1. The dynamic analysis of the correlation of light factors to quality traits. The correlation efficiency of light factors (solar radiation, Lux meter, and light hours) to (a) protein content, (b) amylose content, (c), alkali consumption (d) grain length, (e) brown rice ratio, and (f) head rice ratio. **Figure S2.** The dynamic analysis of the correlation of humidity factors to quality traits. The correlation efficiency of humidity factors (day average humidity and night average humidity) to (a) protein content, (b) amylose content, (c) alkali consumption, (d) grain length, (e) brown rice ratio, and (f) head rice ratio. **Table S1.** The days to heading after sowing of 155 lines at four areas in 2015 and 2016. **Table S2.** The fertility of soil in four areas. **Table S3.** The data of rice quality in 2015 and 2016. **Table S4.** Path analysis of the effect of environmental factors on rice quality. (PPTX 472 kb)
