# Supplementary material for: Deciphering the Environmental Impacts on Rice Quality for Different Rice Cultivated Areas
Source: Rice (N Y). 2018 Jan 19;11:7. doi: 10.1186/s12284-018-0198-1 (PMC5775188; doi:10.1186/s12284-018-0198-1)
Supplement: Additional file 1: Figure S1. — The dynamic analysis of the correlation of light factors to quality traits. The correlation efficiency of light factors (solar radiation, Lux meter, and light hours) to (a) protein content, (b) amylose content, (c), alkali consumption (d) grain length, (e) brown rice ratio, and (f) head rice ratio. Figure S2. The dynamic analysis of the correlation of humidity factors to quality traits. The correlation efficiency of humidity factors (day average humidity and night average humidity) to (a) protein content, (b) amylose content, (c) alkali consumption, (d) grain length, (e) brown rice ratio, and (f) head rice ratio. Table S1. The days to heading after sowing of 155 lines at four areas in 2015 and 2016. Table S2. The fertility of soil in four areas. Table S3. The data of rice quality in 2015 and 2016. Table S4. Path analysis of the effect of environmental factors on rice quality. (PPTX 472 kb) [file 12284_2018_198_MOESM1_ESM.pptx]

## Slide 1
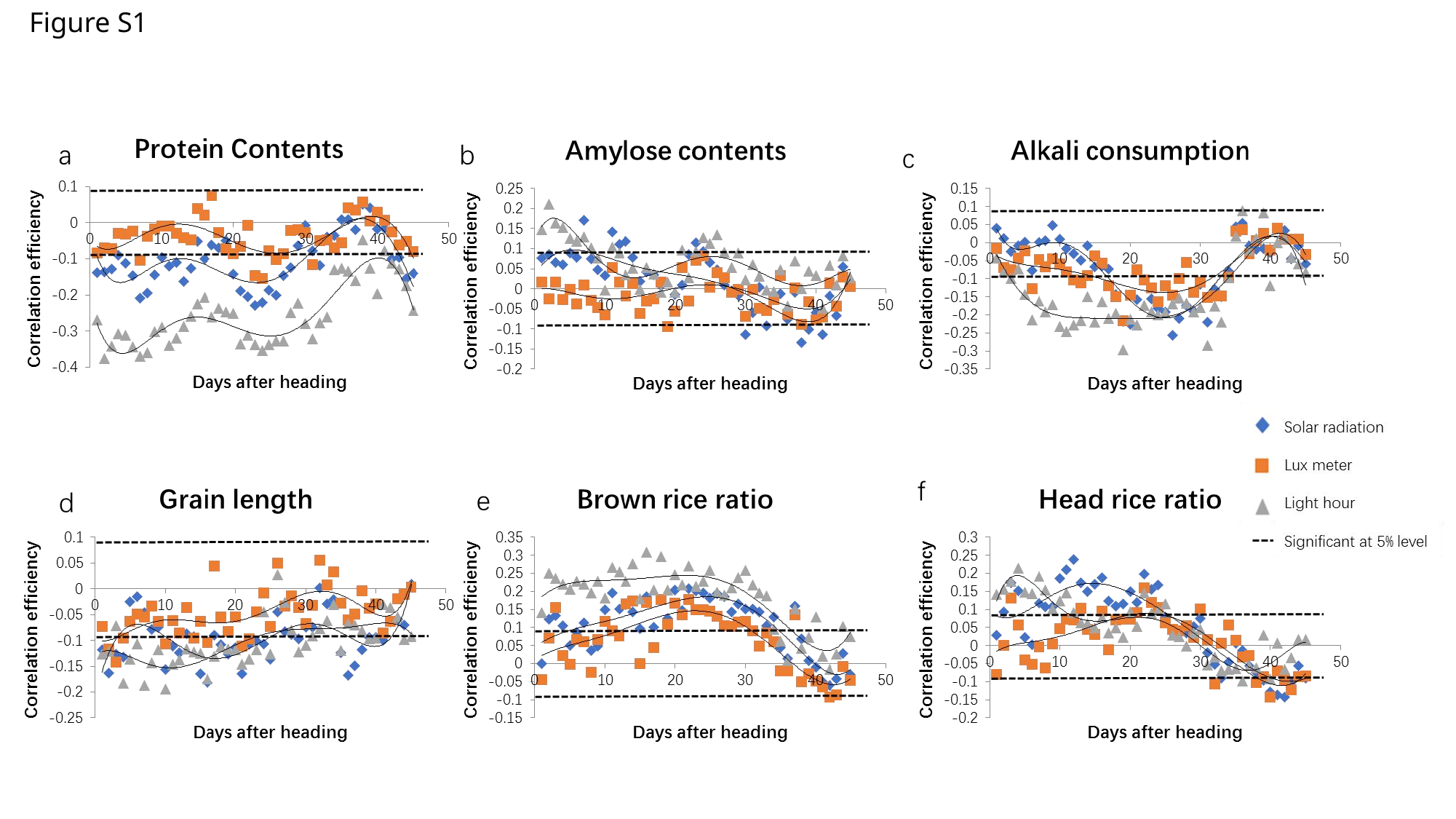

Figure S1

## Slide 2
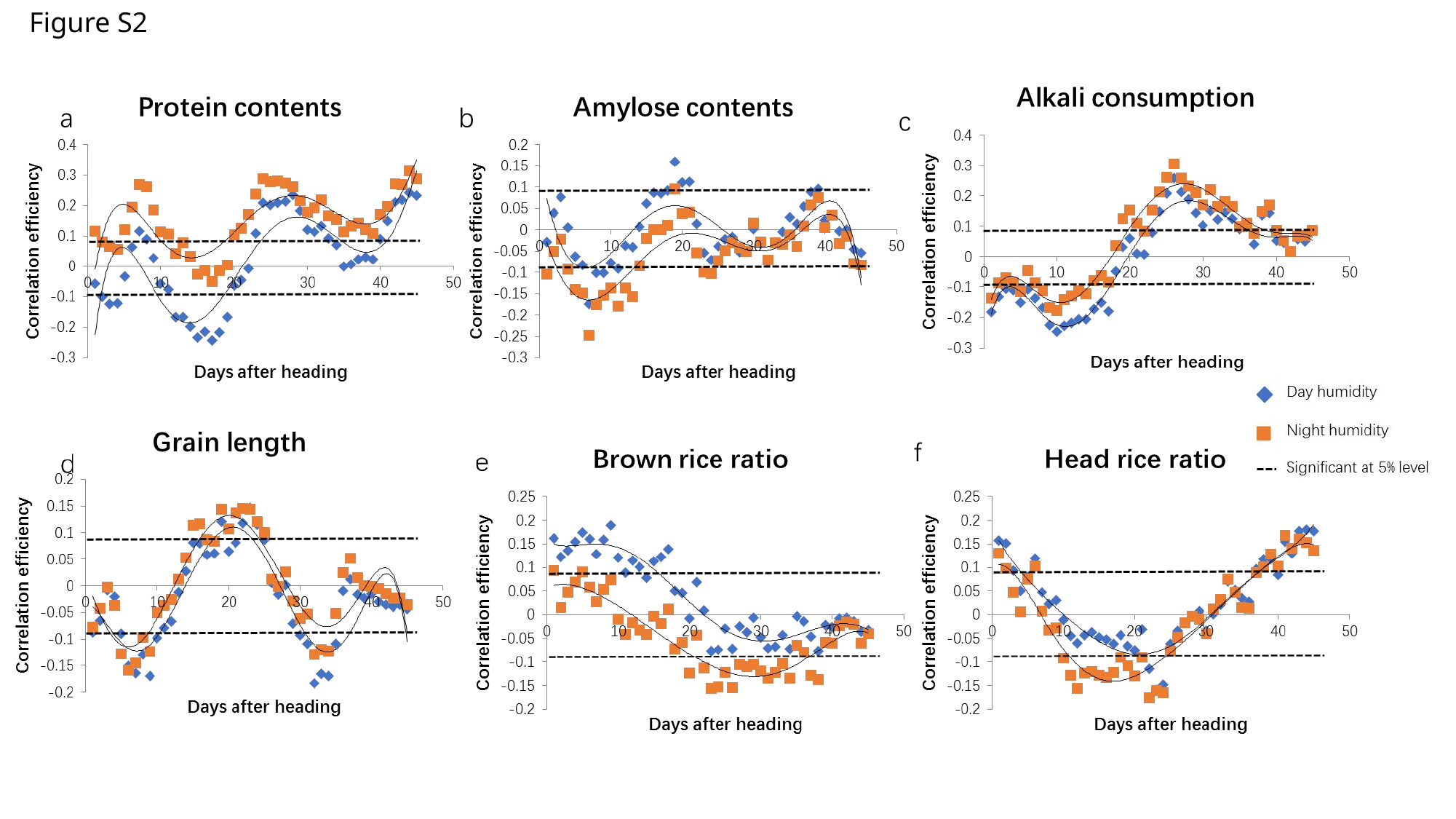

Figure S2

## Slide 3
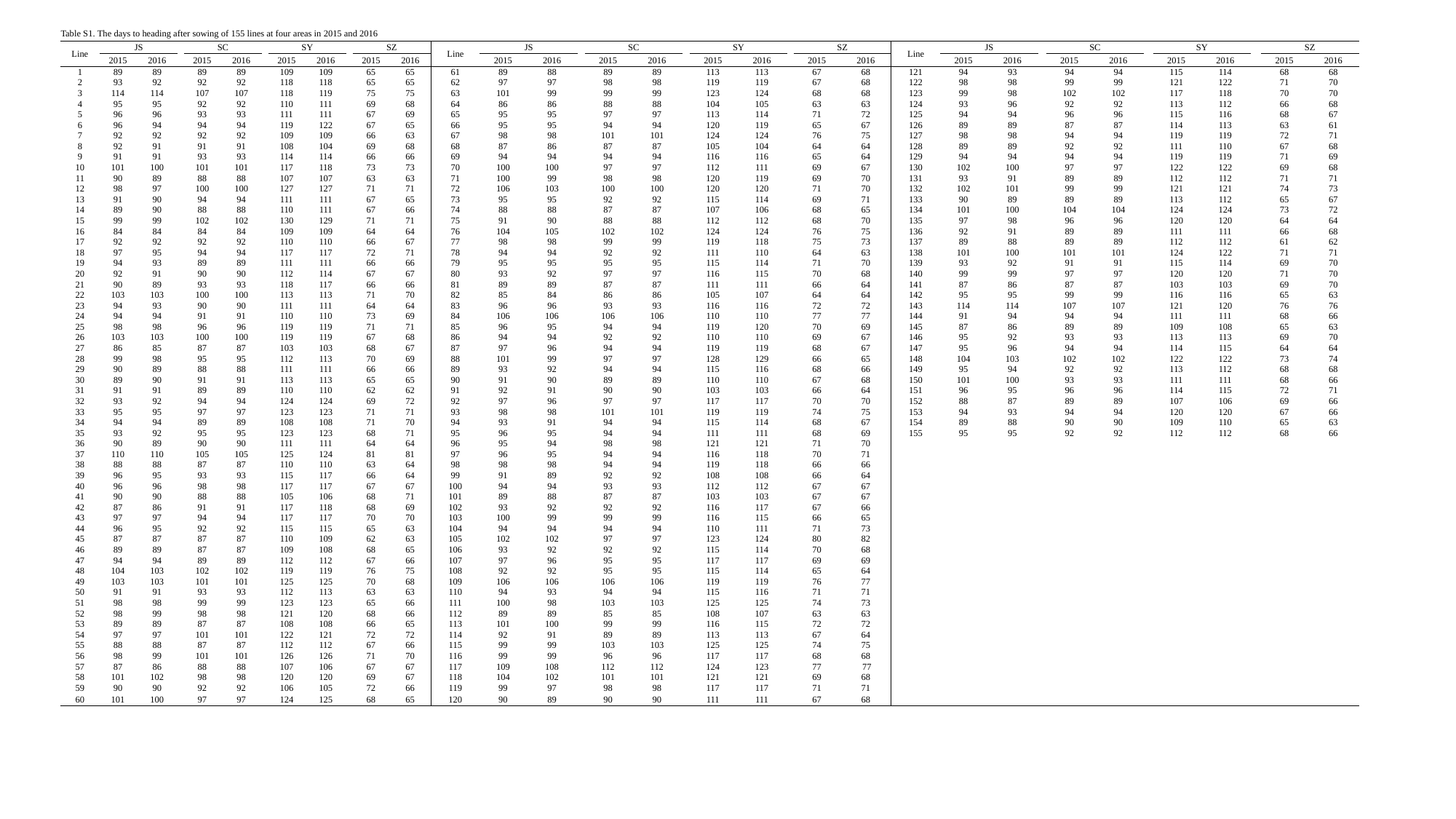

| Table S1. The days to heading after sowing of 155 lines at four areas in 2015 and 2016 | | | | | | | | | | | | | | | | | | | | | | | | | | | | | | | | | | | |
| --- | --- | --- | --- | --- | --- | --- | --- | --- | --- | --- | --- | --- | --- | --- | --- | --- | --- | --- | --- | --- | --- | --- | --- | --- | --- | --- | --- | --- | --- | --- | --- | --- | --- | --- | --- |
| Line | JS | | | SC | | | SY | | | SZ | | Line | JS | | | SC | | | SY | | | SZ | | Line | JS | | | SC | | | SY | | | SZ | |
| | 2015 | 2016 | | 2015 | 2016 | | 2015 | 2016 | | 2015 | 2016 | | 2015 | 2016 | | 2015 | 2016 | | 2015 | 2016 | | 2015 | 2016 | | 2015 | 2016 | | 2015 | 2016 | | 2015 | 2016 | | 2015 | 2016 |
| 1 | 89 | 89 | | 89 | 89 | | 109 | 109 | | 65 | 65 | 61 | 89 | 88 | | 89 | 89 | | 113 | 113 | | 67 | 68 | 121 | 94 | 93 | | 94 | 94 | | 115 | 114 | | 68 | 68 |
| 2 | 93 | 92 | | 92 | 92 | | 118 | 118 | | 65 | 65 | 62 | 97 | 97 | | 98 | 98 | | 119 | 119 | | 67 | 68 | 122 | 98 | 98 | | 99 | 99 | | 121 | 122 | | 71 | 70 |
| 3 | 114 | 114 | | 107 | 107 | | 118 | 119 | | 75 | 75 | 63 | 101 | 99 | | 99 | 99 | | 123 | 124 | | 68 | 68 | 123 | 99 | 98 | | 102 | 102 | | 117 | 118 | | 70 | 70 |
| 4 | 95 | 95 | | 92 | 92 | | 110 | 111 | | 69 | 68 | 64 | 86 | 86 | | 88 | 88 | | 104 | 105 | | 63 | 63 | 124 | 93 | 96 | | 92 | 92 | | 113 | 112 | | 66 | 68 |
| 5 | 96 | 96 | | 93 | 93 | | 111 | 111 | | 67 | 69 | 65 | 95 | 95 | | 97 | 97 | | 113 | 114 | | 71 | 72 | 125 | 94 | 94 | | 96 | 96 | | 115 | 116 | | 68 | 67 |
| 6 | 96 | 94 | | 94 | 94 | | 119 | 122 | | 67 | 65 | 66 | 95 | 95 | | 94 | 94 | | 120 | 119 | | 65 | 67 | 126 | 89 | 89 | | 87 | 87 | | 114 | 113 | | 63 | 61 |
| 7 | 92 | 92 | | 92 | 92 | | 109 | 109 | | 66 | 63 | 67 | 98 | 98 | | 101 | 101 | | 124 | 124 | | 76 | 75 | 127 | 98 | 98 | | 94 | 94 | | 119 | 119 | | 72 | 71 |
| 8 | 92 | 91 | | 91 | 91 | | 108 | 104 | | 69 | 68 | 68 | 87 | 86 | | 87 | 87 | | 105 | 104 | | 64 | 64 | 128 | 89 | 89 | | 92 | 92 | | 111 | 110 | | 67 | 68 |
| 9 | 91 | 91 | | 93 | 93 | | 114 | 114 | | 66 | 66 | 69 | 94 | 94 | | 94 | 94 | | 116 | 116 | | 65 | 64 | 129 | 94 | 94 | | 94 | 94 | | 119 | 119 | | 71 | 69 |
| 10 | 101 | 100 | | 101 | 101 | | 117 | 118 | | 73 | 73 | 70 | 100 | 100 | | 97 | 97 | | 112 | 111 | | 69 | 67 | 130 | 102 | 100 | | 97 | 97 | | 122 | 122 | | 69 | 68 |
| 11 | 90 | 89 | | 88 | 88 | | 107 | 107 | | 63 | 63 | 71 | 100 | 99 | | 98 | 98 | | 120 | 119 | | 69 | 70 | 131 | 93 | 91 | | 89 | 89 | | 112 | 112 | | 71 | 71 |
| 12 | 98 | 97 | | 100 | 100 | | 127 | 127 | | 71 | 71 | 72 | 106 | 103 | | 100 | 100 | | 120 | 120 | | 71 | 70 | 132 | 102 | 101 | | 99 | 99 | | 121 | 121 | | 74 | 73 |
| 13 | 91 | 90 | | 94 | 94 | | 111 | 111 | | 67 | 65 | 73 | 95 | 95 | | 92 | 92 | | 115 | 114 | | 69 | 71 | 133 | 90 | 89 | | 89 | 89 | | 113 | 112 | | 65 | 67 |
| 14 | 89 | 90 | | 88 | 88 | | 110 | 111 | | 67 | 66 | 74 | 88 | 88 | | 87 | 87 | | 107 | 106 | | 68 | 65 | 134 | 101 | 100 | | 104 | 104 | | 124 | 124 | | 73 | 72 |
| 15 | 99 | 99 | | 102 | 102 | | 130 | 129 | | 71 | 71 | 75 | 91 | 90 | | 88 | 88 | | 112 | 112 | | 68 | 70 | 135 | 97 | 98 | | 96 | 96 | | 120 | 120 | | 64 | 64 |
| 16 | 84 | 84 | | 84 | 84 | | 109 | 109 | | 64 | 64 | 76 | 104 | 105 | | 102 | 102 | | 124 | 124 | | 76 | 75 | 136 | 92 | 91 | | 89 | 89 | | 111 | 111 | | 66 | 68 |
| 17 | 92 | 92 | | 92 | 92 | | 110 | 110 | | 66 | 67 | 77 | 98 | 98 | | 99 | 99 | | 119 | 118 | | 75 | 73 | 137 | 89 | 88 | | 89 | 89 | | 112 | 112 | | 61 | 62 |
| 18 | 97 | 95 | | 94 | 94 | | 117 | 117 | | 72 | 71 | 78 | 94 | 94 | | 92 | 92 | | 111 | 110 | | 64 | 63 | 138 | 101 | 100 | | 101 | 101 | | 124 | 122 | | 71 | 71 |
| 19 | 94 | 93 | | 89 | 89 | | 111 | 111 | | 66 | 66 | 79 | 95 | 95 | | 95 | 95 | | 115 | 114 | | 71 | 70 | 139 | 93 | 92 | | 91 | 91 | | 115 | 114 | | 69 | 70 |
| 20 | 92 | 91 | | 90 | 90 | | 112 | 114 | | 67 | 67 | 80 | 93 | 92 | | 97 | 97 | | 116 | 115 | | 70 | 68 | 140 | 99 | 99 | | 97 | 97 | | 120 | 120 | | 71 | 70 |
| 21 | 90 | 89 | | 93 | 93 | | 118 | 117 | | 66 | 66 | 81 | 89 | 89 | | 87 | 87 | | 111 | 111 | | 66 | 64 | 141 | 87 | 86 | | 87 | 87 | | 103 | 103 | | 69 | 70 |
| 22 | 103 | 103 | | 100 | 100 | | 113 | 113 | | 71 | 70 | 82 | 85 | 84 | | 86 | 86 | | 105 | 107 | | 64 | 64 | 142 | 95 | 95 | | 99 | 99 | | 116 | 116 | | 65 | 63 |
| 23 | 94 | 93 | | 90 | 90 | | 111 | 111 | | 64 | 64 | 83 | 96 | 96 | | 93 | 93 | | 116 | 116 | | 72 | 72 | 143 | 114 | 114 | | 107 | 107 | | 121 | 120 | | 76 | 76 |
| 24 | 94 | 94 | | 91 | 91 | | 110 | 110 | | 73 | 69 | 84 | 106 | 106 | | 106 | 106 | | 110 | 110 | | 77 | 77 | 144 | 91 | 94 | | 94 | 94 | | 111 | 111 | | 68 | 66 |
| 25 | 98 | 98 | | 96 | 96 | | 119 | 119 | | 71 | 71 | 85 | 96 | 95 | | 94 | 94 | | 119 | 120 | | 70 | 69 | 145 | 87 | 86 | | 89 | 89 | | 109 | 108 | | 65 | 63 |
| 26 | 103 | 103 | | 100 | 100 | | 119 | 119 | | 67 | 68 | 86 | 94 | 94 | | 92 | 92 | | 110 | 110 | | 69 | 67 | 146 | 95 | 92 | | 93 | 93 | | 113 | 113 | | 69 | 70 |
| 27 | 86 | 85 | | 87 | 87 | | 103 | 103 | | 68 | 67 | 87 | 97 | 96 | | 94 | 94 | | 119 | 119 | | 68 | 67 | 147 | 95 | 96 | | 94 | 94 | | 114 | 115 | | 64 | 64 |
| 28 | 99 | 98 | | 95 | 95 | | 112 | 113 | | 70 | 69 | 88 | 101 | 99 | | 97 | 97 | | 128 | 129 | | 66 | 65 | 148 | 104 | 103 | | 102 | 102 | | 122 | 122 | | 73 | 74 |
| 29 | 90 | 89 | | 88 | 88 | | 111 | 111 | | 66 | 66 | 89 | 93 | 92 | | 94 | 94 | | 115 | 116 | | 68 | 66 | 149 | 95 | 94 | | 92 | 92 | | 113 | 112 | | 68 | 68 |
| 30 | 89 | 90 | | 91 | 91 | | 113 | 113 | | 65 | 65 | 90 | 91 | 90 | | 89 | 89 | | 110 | 110 | | 67 | 68 | 150 | 101 | 100 | | 93 | 93 | | 111 | 111 | | 68 | 66 |
| 31 | 91 | 91 | | 89 | 89 | | 110 | 110 | | 62 | 62 | 91 | 92 | 91 | | 90 | 90 | | 103 | 103 | | 66 | 64 | 151 | 96 | 95 | | 96 | 96 | | 114 | 115 | | 72 | 71 |
| 32 | 93 | 92 | | 94 | 94 | | 124 | 124 | | 69 | 72 | 92 | 97 | 96 | | 97 | 97 | | 117 | 117 | | 70 | 70 | 152 | 88 | 87 | | 89 | 89 | | 107 | 106 | | 69 | 66 |
| 33 | 95 | 95 | | 97 | 97 | | 123 | 123 | | 71 | 71 | 93 | 98 | 98 | | 101 | 101 | | 119 | 119 | | 74 | 75 | 153 | 94 | 93 | | 94 | 94 | | 120 | 120 | | 67 | 66 |
| 34 | 94 | 94 | | 89 | 89 | | 108 | 108 | | 71 | 70 | 94 | 93 | 91 | | 94 | 94 | | 115 | 114 | | 68 | 67 | 154 | 89 | 88 | | 90 | 90 | | 109 | 110 | | 65 | 63 |
| 35 | 93 | 92 | | 95 | 95 | | 123 | 123 | | 68 | 71 | 95 | 96 | 95 | | 94 | 94 | | 111 | 111 | | 68 | 69 | 155 | 95 | 95 | | 92 | 92 | | 112 | 112 | | 68 | 66 |
| 36 | 90 | 89 | | 90 | 90 | | 111 | 111 | | 64 | 64 | 96 | 95 | 94 | | 98 | 98 | | 121 | 121 | | 71 | 70 | | | | | | | | | | | | |
| 37 | 110 | 110 | | 105 | 105 | | 125 | 124 | | 81 | 81 | 97 | 96 | 95 | | 94 | 94 | | 116 | 118 | | 70 | 71 | | | | | | | | | | | | |
| 38 | 88 | 88 | | 87 | 87 | | 110 | 110 | | 63 | 64 | 98 | 98 | 98 | | 94 | 94 | | 119 | 118 | | 66 | 66 | | | | | | | | | | | | |
| 39 | 96 | 95 | | 93 | 93 | | 115 | 117 | | 66 | 64 | 99 | 91 | 89 | | 92 | 92 | | 108 | 108 | | 66 | 64 | | | | | | | | | | | | |
| 40 | 96 | 96 | | 98 | 98 | | 117 | 117 | | 67 | 67 | 100 | 94 | 94 | | 93 | 93 | | 112 | 112 | | 67 | 67 | | | | | | | | | | | | |
| 41 | 90 | 90 | | 88 | 88 | | 105 | 106 | | 68 | 71 | 101 | 89 | 88 | | 87 | 87 | | 103 | 103 | | 67 | 67 | | | | | | | | | | | | |
| 42 | 87 | 86 | | 91 | 91 | | 117 | 118 | | 68 | 69 | 102 | 93 | 92 | | 92 | 92 | | 116 | 117 | | 67 | 66 | | | | | | | | | | | | |
| 43 | 97 | 97 | | 94 | 94 | | 117 | 117 | | 70 | 70 | 103 | 100 | 99 | | 99 | 99 | | 116 | 115 | | 66 | 65 | | | | | | | | | | | | |
| 44 | 96 | 95 | | 92 | 92 | | 115 | 115 | | 65 | 63 | 104 | 94 | 94 | | 94 | 94 | | 110 | 111 | | 71 | 73 | | | | | | | | | | | | |
| 45 | 87 | 87 | | 87 | 87 | | 110 | 109 | | 62 | 63 | 105 | 102 | 102 | | 97 | 97 | | 123 | 124 | | 80 | 82 | | | | | | | | | | | | |
| 46 | 89 | 89 | | 87 | 87 | | 109 | 108 | | 68 | 65 | 106 | 93 | 92 | | 92 | 92 | | 115 | 114 | | 70 | 68 | | | | | | | | | | | | |
| 47 | 94 | 94 | | 89 | 89 | | 112 | 112 | | 67 | 66 | 107 | 97 | 96 | | 95 | 95 | | 117 | 117 | | 69 | 69 | | | | | | | | | | | | |
| 48 | 104 | 103 | | 102 | 102 | | 119 | 119 | | 76 | 75 | 108 | 92 | 92 | | 95 | 95 | | 115 | 114 | | 65 | 64 | | | | | | | | | | | | |
| 49 | 103 | 103 | | 101 | 101 | | 125 | 125 | | 70 | 68 | 109 | 106 | 106 | | 106 | 106 | | 119 | 119 | | 76 | 77 | | | | | | | | | | | | |
| 50 | 91 | 91 | | 93 | 93 | | 112 | 113 | | 63 | 63 | 110 | 94 | 93 | | 94 | 94 | | 115 | 116 | | 71 | 71 | | | | | | | | | | | | |
| 51 | 98 | 98 | | 99 | 99 | | 123 | 123 | | 65 | 66 | 111 | 100 | 98 | | 103 | 103 | | 125 | 125 | | 74 | 73 | | | | | | | | | | | | |
| 52 | 98 | 99 | | 98 | 98 | | 121 | 120 | | 68 | 66 | 112 | 89 | 89 | | 85 | 85 | | 108 | 107 | | 63 | 63 | | | | | | | | | | | | |
| 53 | 89 | 89 | | 87 | 87 | | 108 | 108 | | 66 | 65 | 113 | 101 | 100 | | 99 | 99 | | 116 | 115 | | 72 | 72 | | | | | | | | | | | | |
| 54 | 97 | 97 | | 101 | 101 | | 122 | 121 | | 72 | 72 | 114 | 92 | 91 | | 89 | 89 | | 113 | 113 | | 67 | 64 | | | | | | | | | | | | |
| 55 | 88 | 88 | | 87 | 87 | | 112 | 112 | | 67 | 66 | 115 | 99 | 99 | | 103 | 103 | | 125 | 125 | | 74 | 75 | | | | | | | | | | | | |
| 56 | 98 | 99 | | 101 | 101 | | 126 | 126 | | 71 | 70 | 116 | 99 | 99 | | 96 | 96 | | 117 | 117 | | 68 | 68 | | | | | | | | | | | | |
| 57 | 87 | 86 | | 88 | 88 | | 107 | 106 | | 67 | 67 | 117 | 109 | 108 | | 112 | 112 | | 124 | 123 | | 77 | 77 | | | | | | | | | | | | |
| 58 | 101 | 102 | | 98 | 98 | | 120 | 120 | | 69 | 67 | 118 | 104 | 102 | | 101 | 101 | | 121 | 121 | | 69 | 68 | | | | | | | | | | | | |
| 59 | 90 | 90 | | 92 | 92 | | 106 | 105 | | 72 | 66 | 119 | 99 | 97 | | 98 | 98 | | 117 | 117 | | 71 | 71 | | | | | | | | | | | | |
| 60 | 101 | 100 | | 97 | 97 | | 124 | 125 | | 68 | 65 | 120 | 90 | 89 | | 90 | 90 | | 111 | 111 | | 67 | 68 | | | | | | | | | | | | |

## Slide 4
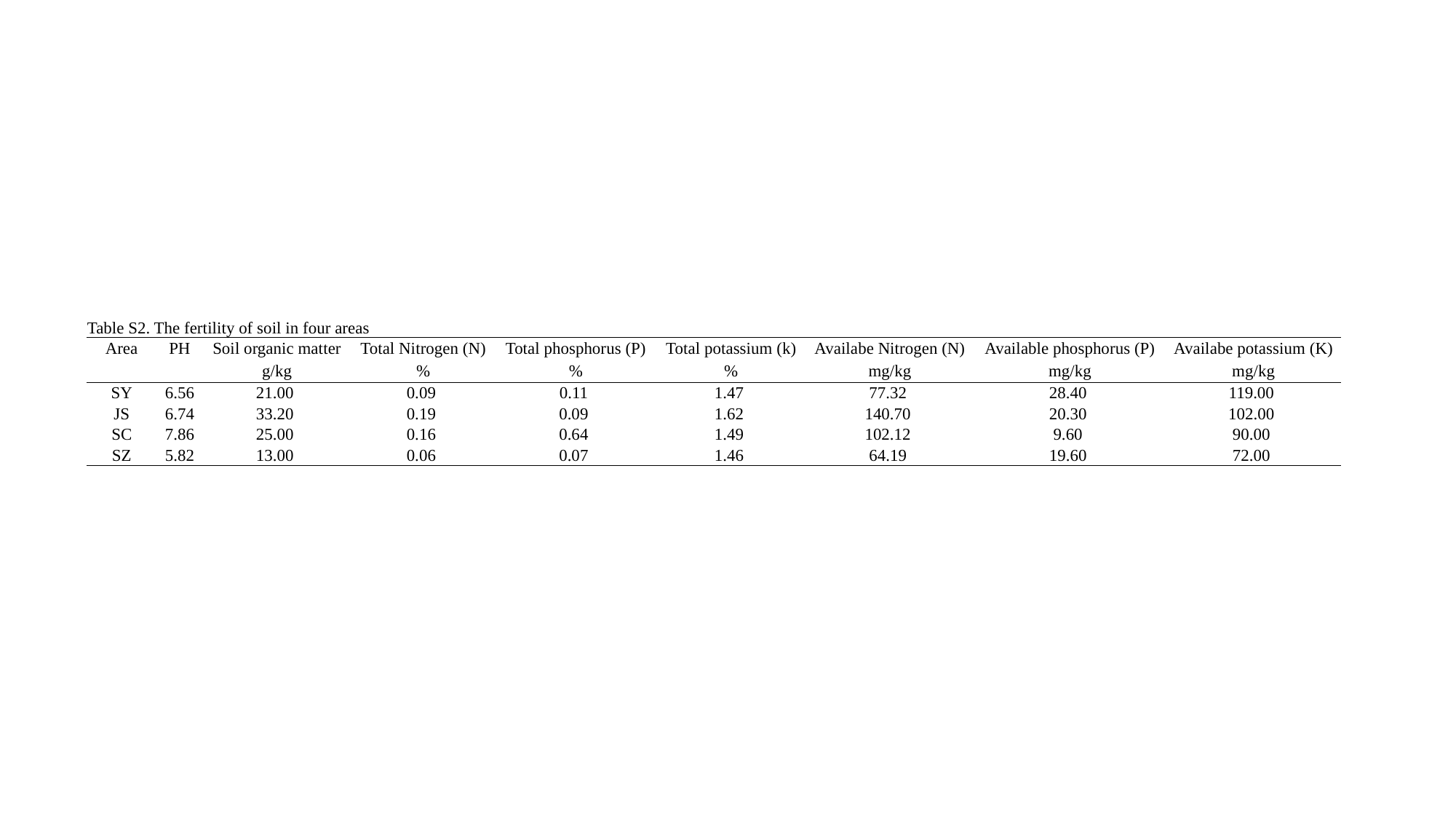

| Table S2. The fertility of soil in four areas | | | | | | | | |
| --- | --- | --- | --- | --- | --- | --- | --- | --- |
| Area | PH | Soil organic matter | Total Nitrogen (N) | Total phosphorus (P) | Total potassium (k) | Availabe Nitrogen (N) | Available phosphorus (P) | Availabe potassium (K) |
| | | g/kg | % | % | % | mg/kg | mg/kg | mg/kg |
| SY | 6.56 | 21.00 | 0.09 | 0.11 | 1.47 | 77.32 | 28.40 | 119.00 |
| JS | 6.74 | 33.20 | 0.19 | 0.09 | 1.62 | 140.70 | 20.30 | 102.00 |
| SC | 7.86 | 25.00 | 0.16 | 0.64 | 1.49 | 102.12 | 9.60 | 90.00 |
| SZ | 5.82 | 13.00 | 0.06 | 0.07 | 1.46 | 64.19 | 19.60 | 72.00 |

## Slide 5
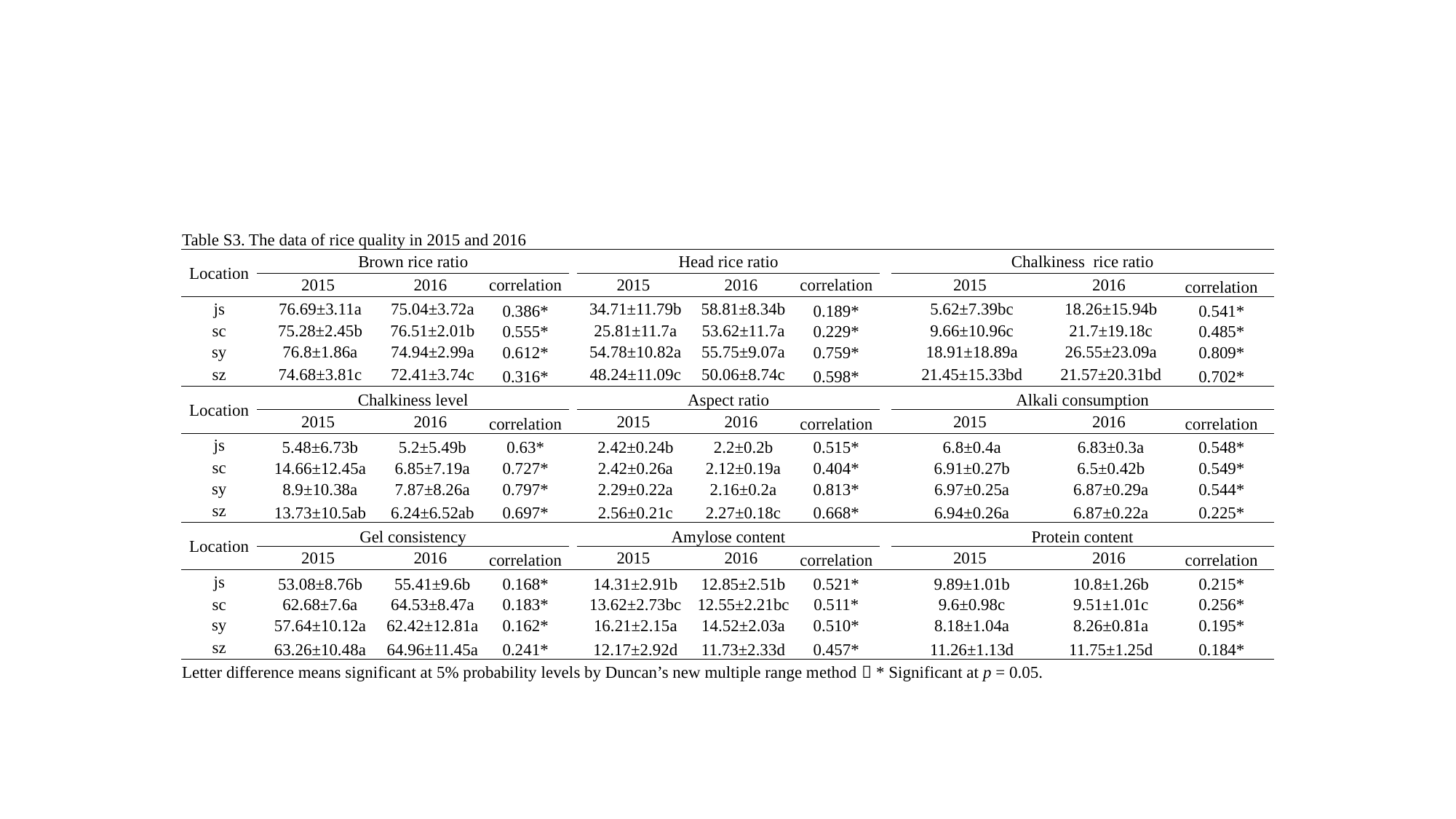

| Table S3. The data of rice quality in 2015 and 2016 | | | | | | | | | | | |
| --- | --- | --- | --- | --- | --- | --- | --- | --- | --- | --- | --- |
| Location | Brown rice ratio | | | | Head rice ratio | | | | Chalkiness rice ratio | | |
| | 2015 | 2016 | correlation | | 2015 | 2016 | correlation | | 2015 | 2016 | correlation |
| js | 76.69±3.11a | 75.04±3.72a | 0.386\* | | 34.71±11.79b | 58.81±8.34b | 0.189\* | | 5.62±7.39bc | 18.26±15.94b | 0.541\* |
| sc | 75.28±2.45b | 76.51±2.01b | 0.555\* | | 25.81±11.7a | 53.62±11.7a | 0.229\* | | 9.66±10.96c | 21.7±19.18c | 0.485\* |
| sy | 76.8±1.86a | 74.94±2.99a | 0.612\* | | 54.78±10.82a | 55.75±9.07a | 0.759\* | | 18.91±18.89a | 26.55±23.09a | 0.809\* |
| sz | 74.68±3.81c | 72.41±3.74c | 0.316\* | | 48.24±11.09c | 50.06±8.74c | 0.598\* | | 21.45±15.33bd | 21.57±20.31bd | 0.702\* |
| Location | Chalkiness level | | | | Aspect ratio | | | | Alkali consumption | | |
| | 2015 | 2016 | correlation | | 2015 | 2016 | correlation | | 2015 | 2016 | correlation |
| js | 5.48±6.73b | 5.2±5.49b | 0.63\* | | 2.42±0.24b | 2.2±0.2b | 0.515\* | | 6.8±0.4a | 6.83±0.3a | 0.548\* |
| sc | 14.66±12.45a | 6.85±7.19a | 0.727\* | | 2.42±0.26a | 2.12±0.19a | 0.404\* | | 6.91±0.27b | 6.5±0.42b | 0.549\* |
| sy | 8.9±10.38a | 7.87±8.26a | 0.797\* | | 2.29±0.22a | 2.16±0.2a | 0.813\* | | 6.97±0.25a | 6.87±0.29a | 0.544\* |
| sz | 13.73±10.5ab | 6.24±6.52ab | 0.697\* | | 2.56±0.21c | 2.27±0.18c | 0.668\* | | 6.94±0.26a | 6.87±0.22a | 0.225\* |
| Location | Gel consistency | | | | Amylose content | | | | Protein content | | |
| | 2015 | 2016 | correlation | | 2015 | 2016 | correlation | | 2015 | 2016 | correlation |
| js | 53.08±8.76b | 55.41±9.6b | 0.168\* | | 14.31±2.91b | 12.85±2.51b | 0.521\* | | 9.89±1.01b | 10.8±1.26b | 0.215\* |
| sc | 62.68±7.6a | 64.53±8.47a | 0.183\* | | 13.62±2.73bc | 12.55±2.21bc | 0.511\* | | 9.6±0.98c | 9.51±1.01c | 0.256\* |
| sy | 57.64±10.12a | 62.42±12.81a | 0.162\* | | 16.21±2.15a | 14.52±2.03a | 0.510\* | | 8.18±1.04a | 8.26±0.81a | 0.195\* |
| sz | 63.26±10.48a | 64.96±11.45a | 0.241\* | | 12.17±2.92d | 11.73±2.33d | 0.457\* | | 11.26±1.13d | 11.75±1.25d | 0.184\* |
| Letter difference means significant at 5% probability levels by Duncan’s new multiple range method，\* Significant at p = 0.05. | | | | | | | | | | | |

## Slide 6
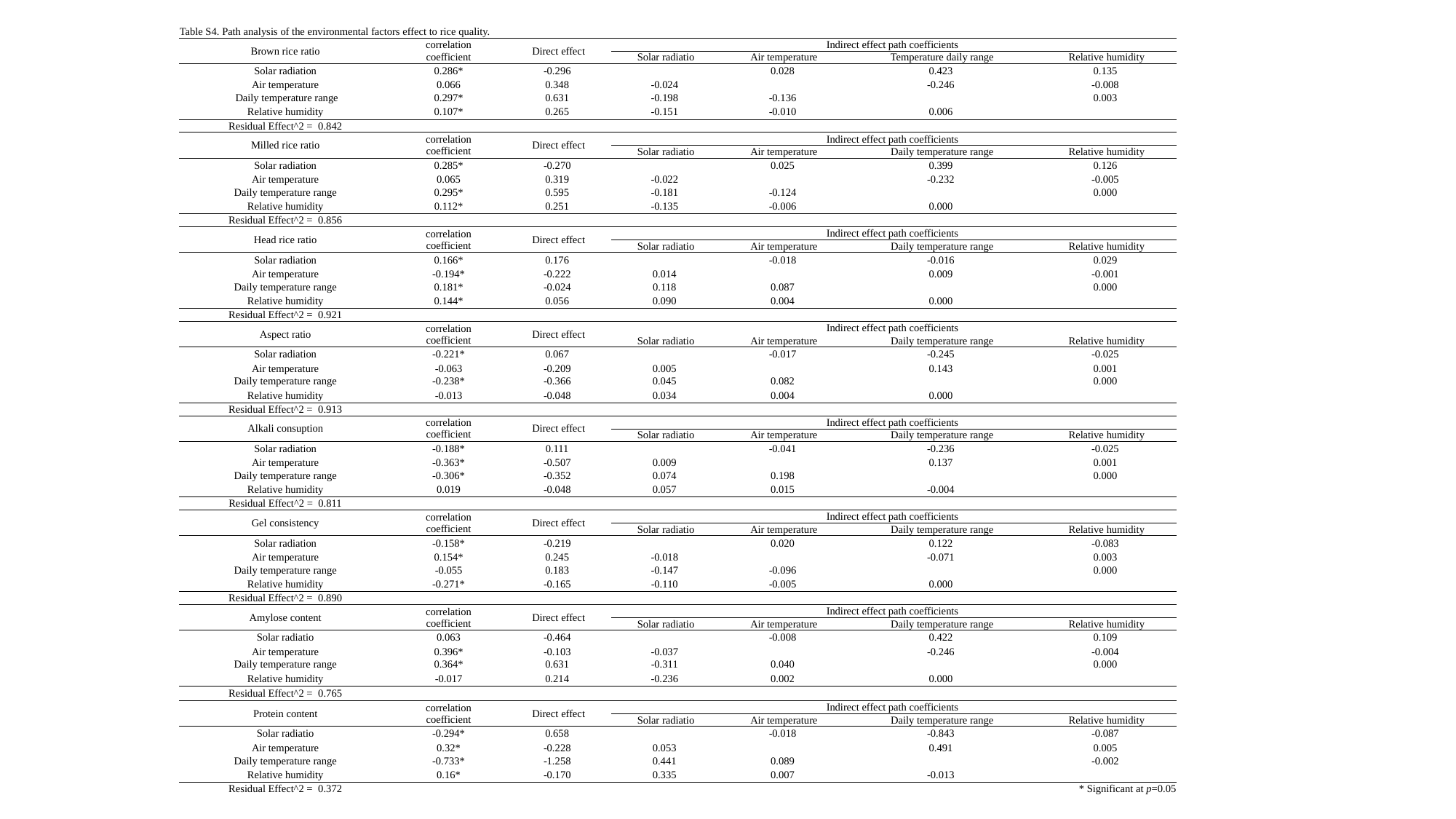

| Table S4. Path analysis of the environmental factors effect to rice quality. | | | | | | |
| --- | --- | --- | --- | --- | --- | --- |
| Brown rice ratio | correlation coefficient | Direct effect | Indirect effect path coefficients | | | |
| | | | Solar radiatio | Air temperature | Temperature daily range | Relative humidity |
| Solar radiation | 0.286\* | -0.296 | | 0.028 | 0.423 | 0.135 |
| Air temperature | 0.066 | 0.348 | -0.024 | | -0.246 | -0.008 |
| Daily temperature range | 0.297\* | 0.631 | -0.198 | -0.136 | | 0.003 |
| Relative humidity | 0.107\* | 0.265 | -0.151 | -0.010 | 0.006 | |
| Residual Effect^2 = 0.842 | | | | | | |
| Milled rice ratio | correlation coefficient | Direct effect | Indirect effect path coefficients | | | |
| | | | Solar radiatio | Air temperature | Daily temperature range | Relative humidity |
| Solar radiation | 0.285\* | -0.270 | | 0.025 | 0.399 | 0.126 |
| Air temperature | 0.065 | 0.319 | -0.022 | | -0.232 | -0.005 |
| Daily temperature range | 0.295\* | 0.595 | -0.181 | -0.124 | | 0.000 |
| Relative humidity | 0.112\* | 0.251 | -0.135 | -0.006 | 0.000 | |
| Residual Effect^2 = 0.856 | | | | | | |
| Head rice ratio | correlation coefficient | Direct effect | Indirect effect path coefficients | | | |
| | | | Solar radiatio | Air temperature | Daily temperature range | Relative humidity |
| Solar radiation | 0.166\* | 0.176 | | -0.018 | -0.016 | 0.029 |
| Air temperature | -0.194\* | -0.222 | 0.014 | | 0.009 | -0.001 |
| Daily temperature range | 0.181\* | -0.024 | 0.118 | 0.087 | | 0.000 |
| Relative humidity | 0.144\* | 0.056 | 0.090 | 0.004 | 0.000 | |
| Residual Effect^2 = 0.921 | | | | | | |
| Aspect ratio | correlation coefficient | Direct effect | Indirect effect path coefficients | | | |
| | | | Solar radiatio | Air temperature | Daily temperature range | Relative humidity |
| Solar radiation | -0.221\* | 0.067 | | -0.017 | -0.245 | -0.025 |
| Air temperature | -0.063 | -0.209 | 0.005 | | 0.143 | 0.001 |
| Daily temperature range | -0.238\* | -0.366 | 0.045 | 0.082 | | 0.000 |
| Relative humidity | -0.013 | -0.048 | 0.034 | 0.004 | 0.000 | |
| Residual Effect^2 = 0.913 | | | | | | |
| Alkali consuption | correlation coefficient | Direct effect | Indirect effect path coefficients | | | |
| | | | Solar radiatio | Air temperature | Daily temperature range | Relative humidity |
| Solar radiation | -0.188\* | 0.111 | | -0.041 | -0.236 | -0.025 |
| Air temperature | -0.363\* | -0.507 | 0.009 | | 0.137 | 0.001 |
| Daily temperature range | -0.306\* | -0.352 | 0.074 | 0.198 | | 0.000 |
| Relative humidity | 0.019 | -0.048 | 0.057 | 0.015 | -0.004 | |
| Residual Effect^2 = 0.811 | | | | | | |
| Gel consistency | correlation coefficient | Direct effect | Indirect effect path coefficients | | | |
| | | | Solar radiatio | Air temperature | Daily temperature range | Relative humidity |
| Solar radiation | -0.158\* | -0.219 | | 0.020 | 0.122 | -0.083 |
| Air temperature | 0.154\* | 0.245 | -0.018 | | -0.071 | 0.003 |
| Daily temperature range | -0.055 | 0.183 | -0.147 | -0.096 | | 0.000 |
| Relative humidity | -0.271\* | -0.165 | -0.110 | -0.005 | 0.000 | |
| Residual Effect^2 = 0.890 | | | | | | |
| Amylose content | correlation coefficient | Direct effect | Indirect effect path coefficients | | | |
| | | | Solar radiatio | Air temperature | Daily temperature range | Relative humidity |
| Solar radiatio | 0.063 | -0.464 | | -0.008 | 0.422 | 0.109 |
| Air temperature | 0.396\* | -0.103 | -0.037 | | -0.246 | -0.004 |
| Daily temperature range | 0.364\* | 0.631 | -0.311 | 0.040 | | 0.000 |
| Relative humidity | -0.017 | 0.214 | -0.236 | 0.002 | 0.000 | |
| Residual Effect^2 = 0.765 | | | | | | |
| Protein content | correlation coefficient | Direct effect | Indirect effect path coefficients | | | |
| | | | Solar radiatio | Air temperature | Daily temperature range | Relative humidity |
| Solar radiatio | -0.294\* | 0.658 | | -0.018 | -0.843 | -0.087 |
| Air temperature | 0.32\* | -0.228 | 0.053 | | 0.491 | 0.005 |
| Daily temperature range | -0.733\* | -1.258 | 0.441 | 0.089 | | -0.002 |
| Relative humidity | 0.16\* | -0.170 | 0.335 | 0.007 | -0.013 | |
| Residual Effect^2 = 0.372 | | | | | \* Significant at p=0.05 | |
